# Cellular and Molecular Aspects of Anti-Melanoma Effect of Minocycline—A Study of Cytotoxicity and Apoptosis on Human Melanotic Melanoma Cells

**DOI:** 10.3390/ijms21186917

**Published:** 2020-09-21

**Authors:** Jakub Rok, Zuzanna Rzepka, Artur Beberok, Justyna Pawlik, Dorota Wrześniok

**Affiliations:** Department of Pharmaceutical Chemistry, Faculty of Pharmaceutical Sciences in Sosnowiec, Medical University of Silesia, Katowice, Jagiellońska 4, 41-200 Sosnowiec, Poland; zrzepka@sum.edu.pl (Z.R.); abeberok@sum.edu.pl (A.B.); d200677@365.sum.edu.pl (J.P.); dwrzesniok@sum.edu.pl (D.W.)

**Keywords:** minocycline, melanoma, apoptosis, caspases, cell cycle, DNA fragmentation

## Abstract

Minocycline is a tetracycline compound with pleiotropic pharmacological properties. In addition to its antibacterial action, it shows many non-antimicrobial effects, including an anti-cancer activity. The anti-cancer action was confirmed in studies on ovarian carcinoma cells, hepatocellular carcinoma cells, glioma cells, or acute myeloid leukemia cells. Malignant melanoma remains a serious medical problem despite the extensive knowledge of the disease. The low effectiveness of the standard treatment, as well as the resistance to therapy, result in high mortality rates. This work aimed to investigate the potential and mechanisms of anti-melanoma action of minocycline. Human skin melanotic melanoma cell line COLO 829 was used in the study. The obtained results showed that minocycline decreased cell viability and inhibited the growth of melanoma cells, proportional to the drug concentration as well as to the time of incubation. The EC_50_ values were calculated to be 78.6 µM, 31.7 µM, and 13.9 µM for 24 h, 48 h, and 72 h, respectively. It was observed that treated cells had a disturbed cell cycle and significantly changed morphology. Moreover, minocycline caused a decrease in mitochondrial membrane potential and an increase in cells with a low level of reduced thiols. Finally, it was found that the anti-melanoma effect of minocycline was related to the induction of apoptosis. The drug activated caspases 8, 9, and 3/7 as well as increased the number of annexin V-positive cells. The presented results show that minocycline possesses anti-melanoma potential.

## 1. Introduction

Melanoma, a word derived from the Greek *melas*, ‘dark’ and *oma* ‘tumor’, is the name of skin cancer, derived from highly specialized, melanin-producing cells, i.e., melanocytes [1]. Despite the large amount of information currently known, melanoma remains a serious medical problem. It was found that the European annual incidence of malignant melanoma ranged from 3–5/100,000 in Mediterranean countries to 12–35/100,000 in Nordic countries, whereas it could reach over 50/100,000 in Australia or New Zealand [2]. Although melanoma occurs less commonly than other skin cancers, it is responsible for nearly 73% of skin cancer-related deaths [3]. 

It was confirmed that cutaneous melanoma cells have a high number of ultraviolet-signature mutations, such as C→T or G→T transitions, caused by UVB and UVA radiation, respectively [4]. These mutations are observed in about 80% of melanomas [5]. Currently, genetic analysis and detected mutations in melanoma cells are used for the prognosis of outcome in melanoma as well as for choosing the best form of therapy [6,7]. The genetic classification plays an important role in making a decision of targeted pharmacotherapy that involves BRAF inhibitors: vemurafenib, dabrafenib, encorafenib, also in combination with MEK inhibitors: trametinib, cobimetinib, binimetinib [8]. Of note, the use of targeted therapy is limited to the mutant subtypes of melanoma. 

Except for the molecular-targeted therapy, the treatment of melanoma involves surgical resection of the tumor and the surrounding healthy tissue—the primary treatment for localized melanoma. After the removal of cancer or in the case of metastasis, the following treatment modalities of melanoma are recommended: radiotherapy, chemotherapy with dacarbazine as well as immunotherapy with interleukin-2 and antibodies acting as immune checkpoint inhibitors: anti-CTLA4—ipilimumab and anti-PD-1—nivolumab, pembrolizumab [9,10]. Although there is a wide variety of melanoma therapies, their relatively low efficiency and limitations prompt searches for new therapeutic methods. 

The relatively low efficacy and safety of many treatment methods remain one of the most important problems in contemporary oncology. Available data indicate that 86% of patients treated with chemotherapy report at least one adverse effect and, in the case of 60% of patients it was, classified as serious [11]. It was found that the average 5-year survival rates for adult cancers in North America ranged from 14% to 56%, which necessitated the use of a combined therapy [12]. In the case of skin melanoma, surgery is the primary treatment. Moreover, standard chemotherapeutics, immunotherapy, and targeted therapy drugs are involved in the cure. Although early diagnosis and treatment of melanoma are associated with a good prognosis, 5-year survival for patients with stage IV cancer is only 19% [13]. The low effectiveness of the standard approach in cancer treatment, as well as the problem of resistance to applied therapy, triggered a need for molecular profiling of patients and complex optimization of therapy using multidrug combinations of customized agents [14,15]. With reference to the above, a lot of new information and clues have been recently published. They concern both genetic prognosis and profiling of melanoma [16,17,18,19], as well as various drugs, often well-known, which could be considered new anti-melanoma agents, e.g., everolimus [20], benserazide [21] or naturally occurring mangiferin [22].

Minocycline is currently one of the most intensely studied tetracycline antibiotics. The pharmacological action of minocycline includes regulation of cell proliferation and apoptosis as well as anti-inflammatory, antioxidant, and neuroprotective activity [23]. Currently, the drug is considered e.g., in the treatment of rheumatoid arthritis [24], sarcoidosis [25], hyperkeratosis [26], multiple sclerosis [27], acute stroke and traumatic brain injury [28,29], chronic pain [30], depressive symptoms [31] and Parkinson’s disease [32]. In addition to the above, there is much evidence indicating the anticancer activity of minocycline [33].

Previously described mechanisms of minocycline anti-cancer action involve reduction of STAT3 phosphorylation, prevention of NF-κB activation, repression of tumor necrosis factor (TNF)α expression or inhibition of matrix metalloproteinases [33,34,35].

With regard to the above considerations, the question arose about the possibility of using minocycline in the treatment of melanoma. The study aimed to assess the anti-melanoma properties of minocycline using the culture of human melanoma cells.

## 2. Results

### 2.1. The Viability of Human Melanoma Cells Exposed to Minocycline

**Figure 1:**
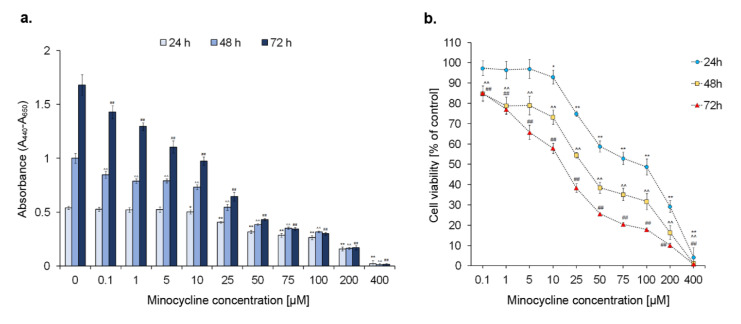
Minocycline inhibits proliferation of melanoma cells (**a**) and reduces their viability (**b**) the screening examination by the WST-1 assay. Cells were incubated with minocycline in concentrations ranging from 0.1 μM to 400 μM for 24 h, 48 h, and 72 h. Mean values ± SD from three independent experiments are presented. * *p* < 0.05, ** *p* < 0.005 vs. control 24 h; ^^ *p* < 0.005 vs. control 48 h; ## *p* < 0.005 vs. control 72 h.

General assessment of the cytotoxic effect of minocycline on melanoma cells was evaluated for the drug concentrations ranging from 0.1 to 400 µM (Figure 1). The test was carried out for 24 h, 48 h, and 72 h. The obtained results indicated that minocycline decreased cell viability and inhibited the growth of the cells during the treatment. The observed effect was proportional to the drug concentration and exposure time. A percentage of viable cells lower than 50% was noticed for 100 µM (ca. 48% of control), 50 µM (ca. 38% of control) and 25 µM (ca. 38% of control) concentrations after 24 h, 48 h, and 72 h, respectively. The highest tested concentration of the drug reduced the percentage of viable cells during the treatment to ca. 1%. The analysis of the absorbance results suggests that minocycline in a concentration up to 25 µM slows down but does not inhibit cell proliferation completely. The significant inhibition of cell division was found for 50 µM and higher concentrations of the drug. Based on the results, the EC_50_ values were calculated to be 78.6 µM, 31.7 µM, and 13.9 µM, following the time of exposure.

Taking into consideration the obtained results, minocycline concentrations of 50 µM, 100 µM, and 200 µM were selected for further analysis. The following experiments were made after 24 h and 48 h-long incubation of human melanoma cells with the tested drug.

### 2.2. The General Evaluation of Human Melanoma Cell Culture Treated with Minocycline

**Figure 2:**
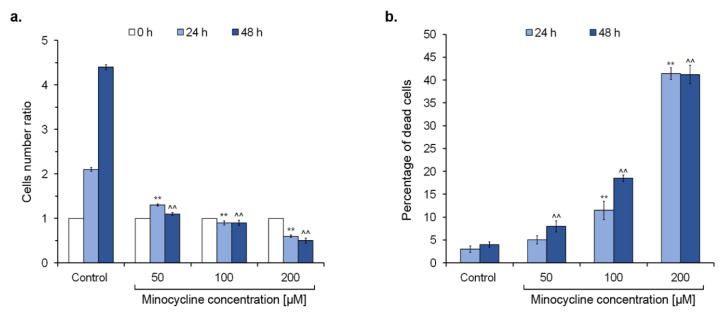
Minocycline inhibits the growth of human melanoma cells and increases the percentage of dead cells after 24 h and 48 h treatment. The cells were cultured with minocycline in a concentration of 50 μM, 100 μM, and 200 μM. The results are presented as the cell number ratio (**a**) and the percentage of dead cells in the tested population (**b**). ** *p* < 0.005 vs. control 24 h; ^^ *p* < 0.005 vs. control 48 h.

**Figure 3:**
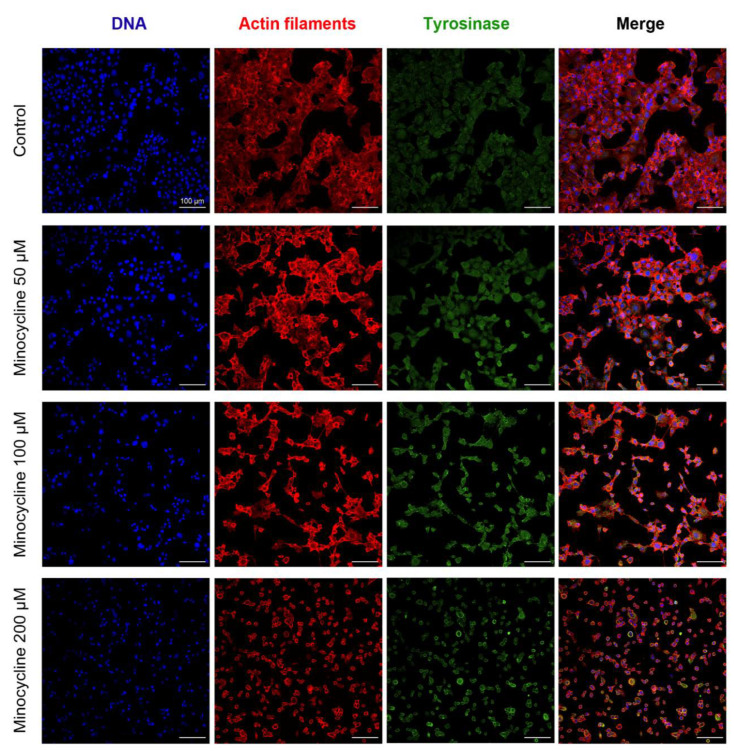
Confocal microscopy imaging of human melanoma cells treated with minocycline in concentrations of 50 μM, 100 μM, and 200 μM. Photographs are presented as separate channels for DNA, actin filaments, and tyrosinase as well as merge images. Scale bar 100 μm.

The culture of human melanoma cells was evaluated after exposure to minocycline. The analysis involved counting cells (Figure 2a), the estimation of dead cells (Figure 2b), as well as confocal microscopy imaging (Figure 3). Comparing the number of cells in each tested culture, it was found that non-treated cells doubled during ca. 24 h and achieved the cell number ratios of 2.1 and 4.4 after 24 h and 48 h-long incubation, respectively. In turn, cells treated with minocycline in the concentrations of 50 µM and 100 µM had a similar ratio value, suggesting the inhibition of cell proliferation. Moreover, the decrease in the cell number in the culture after 24 h and 48 h-long treatments with 200 µM of minocycline was noticed. The observation indicates the probability of cell death during incubation with the highest tested concentration of the drug. The reported changes were reflected in a percentage of DAPI-positive cells. The number of dead cells in the control remained below 5% and increased in treated cultures proportionally to a concentration and the time of incubation in general. The culture exposed to 200 µM of minocycline was characterized by the highest percentage of DAPI-positive cells—ca. 41% after incubation for 24 h and 48 h.

A significant influence of minocycline on human melanoma cells was also noticed in confocal microscope images. The nucleus, actin filaments as well as tyrosinase, one of the specific markers of melanin-producing cells such as melanocytes and melanoma cells, were stained and imaged. The expression of tyrosinase was observed in all samples. Generally, the enzyme was evenly distributed in the cytoplasm. However, in the cells treated with 200 µM of minocycline, tyrosinase tended to accumulate on the periphery of the cytoplasm and around the nucleus. However, this feature can be a result of significant morphological changes. Cells in the control culture showed characteristic morphology and adhered tightly to each other, creating cellular clusters. Minocycline gradually reduced the number of cells and caused their shrinkage. Probably due to the loss of contact, cells exposed to 100 µM of minocycline formed long and thin tabs containing tyrosinase. In turn, all cells treated with 200 µM of the tested drug were round and significantly reduced in size. Their cytoplasm was highly condensed around the nucleus. Moreover, it was observed that cells were located separately or in a few-cell groups in the tested culture.

### 2.3. The Assessment of Human Melanocyte Viability after the Treatment with Minocycline

**Figure 4:**
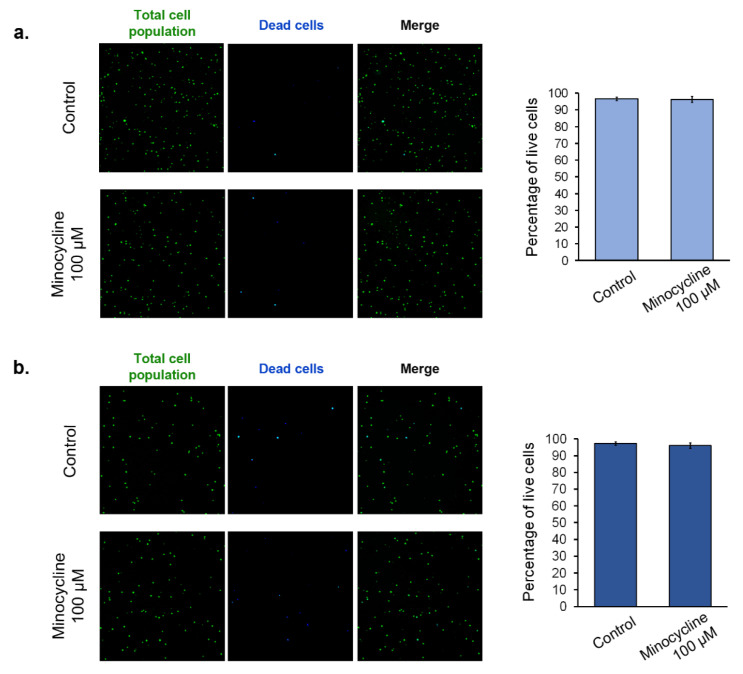
The influence of minocycline on the viability of human normal epidermal melanocytes. Lightly pigmented (**a**) and darkly pigmented (**b**) melanocytes were treated with 100 µM of minocycline for 48 h. Representative pictures of stained, non-fixed cells are presented. The results of the viability are shown as the percentage of live cells in the tested population.

In order to assess the influence of minocycline on normal cells, the viability of human epidermal melanocytes was tested. Lightly pigmented (Figure 4a) and darkly pigmented (Figure 4b) cell lines were used in the study. Due to the two day-long doubling time of the cells, the evaluation was made after the treatment for 48 h. The analysis was based on the non-fixed cell staining with acridine orange (total population) and DAPI (dead cells). The obtained results showed that 100 µM of minocycline did not affect the viability of melanocytes. Simultaneously, significant differences between lightly and darkly pigmented cells were not observed. The percentage of live cells in the tested population in all cases was about 97%. The pictures obtained by the use of the image cytometer showed the presence of only single DAPI-positive cells in the treated as well as non-treated cultures.

### 2.4. Minocycline-Induced Alterations of Human Melanoma Cell Cycle

**Figure 5:**
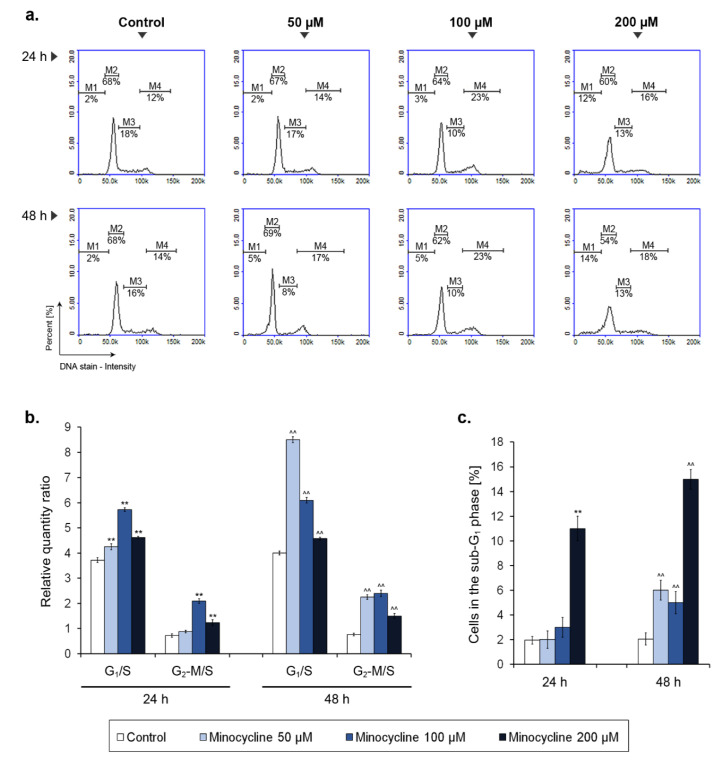
Minocycline-induced changes of cell cycle in human melanoma cells treated for 24 h and 48 h. Representative histograms for the tested groups show distribution of cells in the individual phases of cell cycle (**a**). The results of the cell cycle analysis are presented as a relative quantity ratio of G_1_/S and G_2_-M/S (**b**). The number of cells in the sub-G_1_ phase is shown as the percentage of the tested population (**c**). ** *p* < 0.005 vs. control 24 h; ^^ *p* < 0.005 vs. control 48 h.

The analysis of the melanoma cell cycle was made after 24 h and 48 h incubation with minocycline (Figure 5). The obtained results were also recounted into a relative quantity ratio of G_1_ and G_2_-M phase to S phase. The study showed that minocycline changed the cell cycle profile of human melanoma cells in a manner depending on the incubation time and the drug concentration. In general, minocycline caused an increase in the relative quantity of G_1_/S and G_2_-M/S ratio suggesting an arrest of the cell cycle. The observed alterations were the highest for the concentration of 100 µM after 24 h-long treatment. In turn, after incubation for 48 h, a nearly double increase in G_1_/S ratio was noticed for 50 µM of minocycline. The level of G_2_-M/S ratio after 48 h was similar for 50 µM and 100 µM of the tested drug and was nearly three times higher when compared to the control. The alterations of the ratios resulted mainly from a decrease in the percentage of cells in the S phase as well as the rise of a cell number in the G_2_-M phase. Interestingly, the observed changes in the cell cycle were less severe for the highest concentration of the drug. However, the analysis indicated that 200 µM of minocycline caused a significant elevation in the percentage of cells in the sub-G_1_ phase. The 5- or 7-fold increase was noted when compared to the controls after 24 h and 48 h-long treatments, respectively. The growth of the number of cells in the sub-G_1_ phase was also found for the lower tested concentrations after 48 h.

### 2.5. Minocycline Triggers Melanoma Cell Apoptosis (Annexin V Assay Confirmation)

**Figure 6:**
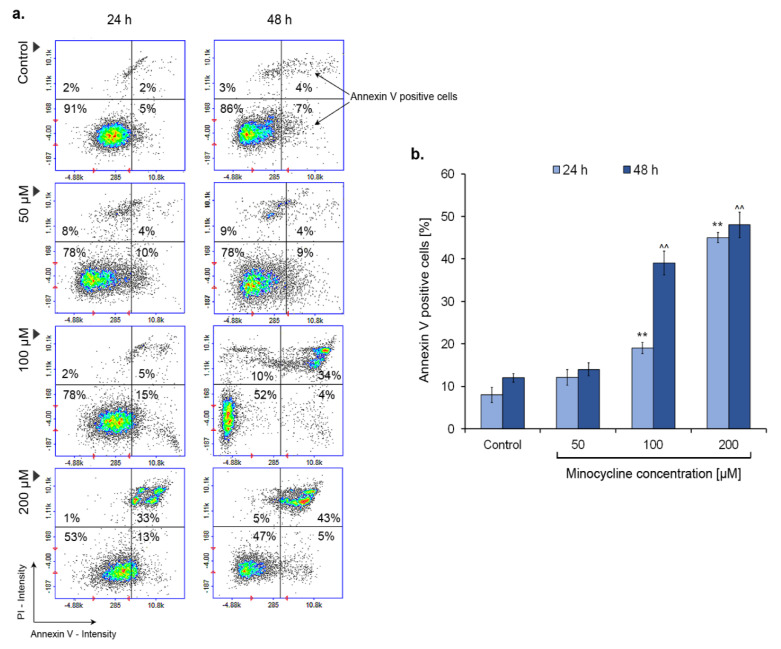
Minocycline induces apoptosis in human melanoma cells—the results of annexin V assay after 24 h and 48 h incubation. Representative scatter plots display a tested cells population divided by a gate into the subpopulations of healthy and apoptotic—annexin V-positive cells (**a**). Mean values of the percentage of annexin V-positive cells are presented in the bar graph (**b**). ** *p* < 0.005 vs. control 24 h; ^^ *p* < 0.005 vs. control 48 h.

The annexin V assay is one of the common tests used to confirm cell apoptosis. In order to examine the process, the melanoma cell culture was incubated with minocycline in concentrations of 50 µM, 100 µM, and 200 µM for 24 h and 48 h. The results presented in Figure 6 indicate that minocycline can induce apoptosis of melanoma cells. The effect was noticeable for samples treated with 100 and 200 µM of minocycline. In the first case, the growth of a percentage of annexin V-positive cells was ca. 10% and ca. 20% for 24 h and 48 h, respectively. In turn, minocycline in a concentration of 200 µM caused an increase in apoptotic cells to ca. 45% and 47% of the tested population.

### 2.6. Minocycline Decreases the Intracellular Level of Reduced Thiols

**Figure 7:**
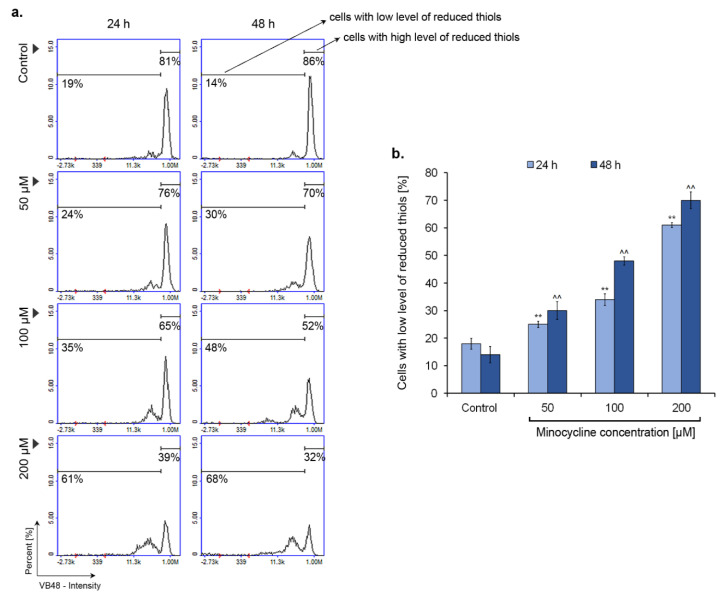
Minocycline decreases the intracellular level of reduced thiols in human melanoma cells after 24 h and 48 h incubation. Individual markers respond to cells with decreased level of reduced thiols and cells with high level of reduced thiols (**a**). Mean values of the percentage of cells with low level of reduced thiols are presented in the bar graph (**b**). ** *p* < 0.005 vs. control 24 h; ^^ *p* < 0.005 vs. control 48 h.

The intracellular level of reduced thiols was examined by the Vitality assay. Investigated melanoma cells were analyzed after the treatment with minocycline in concentrations of 50 µM, 100 µM, and 200 µM for 24 h and 48 h. The obtained results (Figure 7) show that the drug increases the number of cells with a low level of reduced thiols. The effect is proportional to the concentration as well as the time of incubation. Statistically significant changes were noticed already for the 50 µM of minocycline. The increase, in this case, was ca. 15% for 48 h-long treatment. The highest number of cells with a low level of reduced thiols was observed for the concentration of 200 µM—the percentage of cells was ca. 60% and 68% for 24 h and 48 h incubation, respectively. In turn, the largest difference (over 10%) between the 24 h and 48 h incubation times was noticed for 100 µM of minocycline, i.e., ca. 35% and 48%, respectively.

### 2.7. Minocycline Induces DNA Fragmentation in Human Melanoma Cells

**Figure 8:**
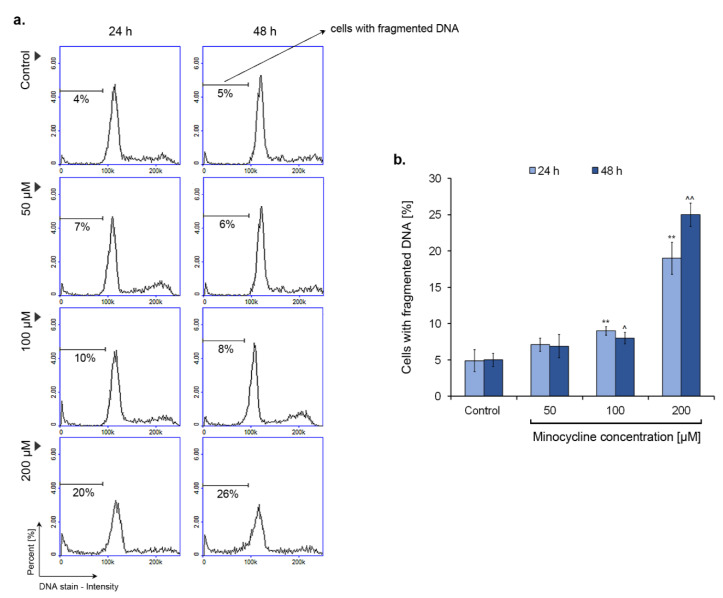
The influence of minocycline on DNA fragmentation in human melanoma cells after 24 h and 48 h treatment. The presented histograms respond to representative results of the experiments (**a**). Mean values of the percentage of cells with fragmented DNA are shown in the bar graph (**b**). ** *p* < 0.005 vs. control 24 h; ^ *p* < 0.05, ^^ *p* < 0.005 vs. control 48 h.

DNA fragmentation is one of the characteristic features of cell apoptosis. The process can be confirmed by the detection of reduced DNA content in tested cells. The principle of this method is leakage of fragmented DNA multimers outside the cell during permeabilization with 70% ethanol. The analysis was performed for cultures of human melanoma cells incubated with 50 µM, 100 µM, and 200 µM of minocycline for 24 h and 48 h. The obtained results (Figure 8) show that the drug induces significantly DNA fragmentation at a concentration of 200 µM. In this case, the percentage of cells with reduced DNA content was ca. 20% and 26% for 24 h and 48 h treatment, respectively. Minocycline in lower concentrations did not cause notable fragmentation of DNA. The increase in cells with fragmented DNA was ca. 4–5% for a concentration of 100 µM.

### 2.8. Minocycline Triggers a Decrease in Mitochondrial Membrane Potential

**Figure 9:**
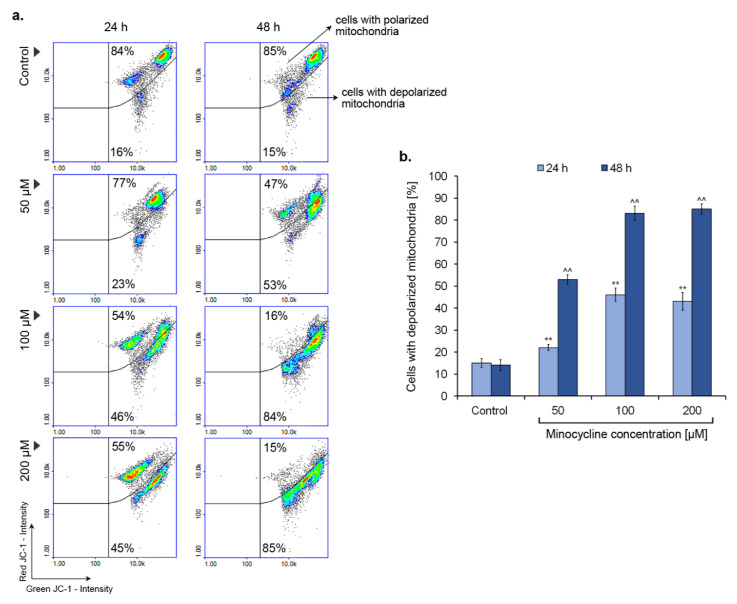
Minocycline decreases mitochondrial membrane potential in human melanoma cells after 24 h and 48 h incubation. Representative scatter plots display a tested cells population divided by a gate to the subpopulations of polarized and depolarized cells (**a**). Mean values of the percentage of cells with lowered mitochondrial membrane potential are presented in the bar graph (**b**). ** *p* < 0.005 vs. control 24 h; ^^ *p* < 0.005 vs. control 48 h.

A decrease in mitochondrial membrane potential is often observed in the early stage of apoptosis. The process is detected by the use of a specific dye JC-1, which can be accumulated in the mitochondrial matrix. The estimation of mitochondrial membrane polarization was performed for human melanoma cells treated with minocycline in concentrations of 50 µM, 100 µM, and 200 µM for 24 h and 48 h (Figure 9). The increase in a number of cells with depolarized mitochondria was observed in all tested samples. Minocycline in a concentration of 50 µM caused a decrease in mitochondrial membrane potential in ca. 23% and 53% of melanoma cells after 24 h and 48 h, respectively. The effect of minocycline on mitochondria polarization was similar for the remaining concentrations. The percentage of cells with depolarized mitochondria after treatment for 24 h and 48 h was ca. 45% and 85%, respectively. In this case, the additional day of incubation resulted in a nearly double augmentation in the observed effect.

### 2.9. Minocycline Activates Caspase 8, 9 and 3/7 in Human Melanoma Cells

**Figure 10:**
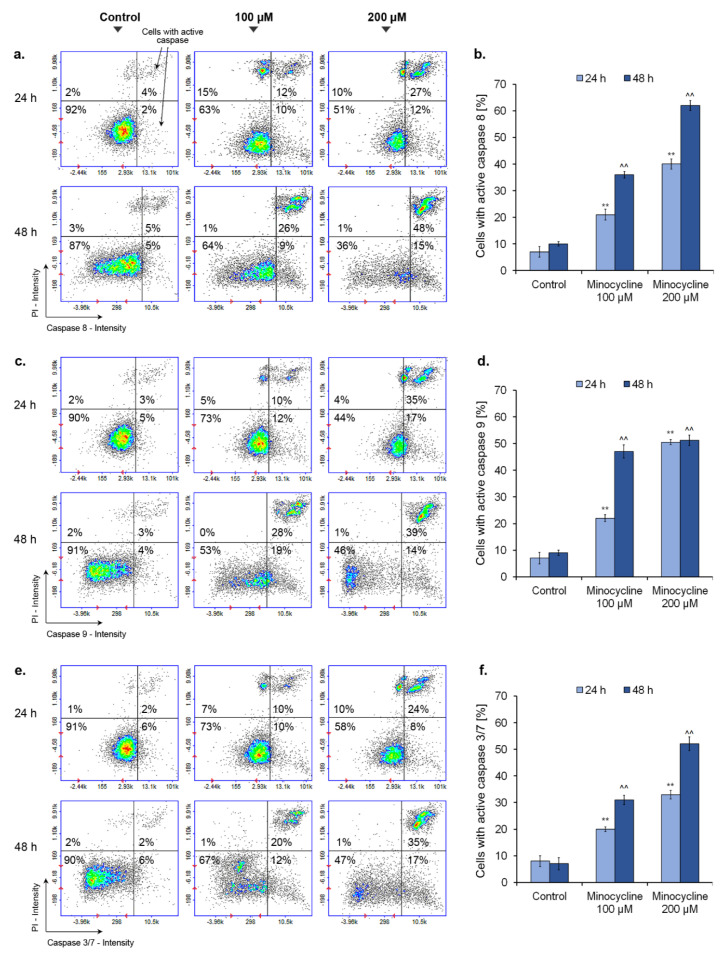
Minocycline activates caspases in human melanoma cells after 24 h and 48 h treatment. Presented scatter plots respond to representative results of the experiments for caspase 8 (**a**), caspase 9 (**c**), and caspase 3/7 (**e**). Mean values of the percentage of cells with activated caspases are presented in the bar graphs (**b**,**d**,**f**). ** *p* < 0.005 vs. control 24 h; ^^ *p* < 0.005 vs. control 48 h.

The activity of caspases was detected during cytometric analysis with the use of caspase-specific fluorochrome-labelled inhibitors. The obtained results, presented in Figure 10, show the increased activity of all tested caspases in melanoma cells treated with minocycline for 24 h and 48 h. The activity growth, in almost all cases, was proportional to the time of incubation as well as to the concentration of the tested drug. Slightly different relationships were observed for caspase 9. A high increase in cells with active caspase 9, ca. 50%, was noticed for 100 µM of minocycline after 48 h as well as for 200 µM of the drug, regardless of the incubation time. After one-day treatment, minocycline in a concentration of 100 µM caused the activation of all caspases in ca. 20% of melanoma cells. The analysis of caspase 8 indicates that the use of a higher concentration of the drug is associated with an almost two-fold increase in the enzyme activity for both incubation times. In turn, the most significant rise of a number of cells with active executioner caspases 3/7 (ca. 52%) was noticed after 48 h treatment with 200 µM of minocycline.

## 3. Discussion

Minocycline is one of the drugs with pleiotropic pharmacological activity. Investigations of minocycline include its potential application in cancer treatment. Some studies show that the drug reduces treatment-related symptoms, such as fatigue and burden in patients with non-small cell lung cancer [36] or erlotinib-related acneiform rash and xerosis in patients with advanced pancreatic cancer [37]. Moreover, there is evidence that minocycline overcomes resistance to topoisomerase inhibition attenuating the efficacy of irinotecan in peritoneal carcinomatosis [38]. Additionally, it acts synergistically with cisplatin in the treatment of hepatocellular carcinoma [39]. In turn, Quinn et al. [34] proved the highly cytotoxic effect of a combination of minocycline with a BH3 mimetic—sabutoclax to pancreatic cancer cells. Anti-proliferative and anti-metastatic properties of minocycline were also confirmed in other studies on various types of cancer, e.g., renal adenocarcinoma [40], breast cancer [41] or malignant gliomas [42]. Additionally, Gilbertson-Beadling et al. [43] showed that minocycline also had anti-angiogenic activity.

The obtained results of our study indicate that minocycline has valuable anti-melanoma properties. The experiments were conducted on human melanotic melanoma cell line—COLO 829. The choice of the cellular model was connected with the ability of tetracyclines to accumulate in melanin-producing cells [44,45]. The process of selective accumulation can be considered a benefit concerning targeted therapy.

Minocycline proved to be a drug inhibiting melanoma cell proliferation and decreasing their viability. EC_50_ values, calculated on the basis of cell viability, were 78.6 µM, 31.7 µM, and 13.9 µM for 24 h, 48 h, and 72 h-long treatment, respectively. It is worth emphasizing that the results indicate the higher anti-melanoma activity of minocycline than of dacarbazine, which is a reference drug in the treatment of melanoma. The study conducted on the same melanoma cells showed that 50 μM and 100 μM of dacarbazine decreased cell viability after 24 h to ca. 58% and 53%, respectively [46].

The anti-melanoma effect of minocycline was also confirmed by the evaluation of the treated culture. The drug significantly reduced the growth of cells as well as increased the number of dead cells during incubation. The changes were also observed in confocal microscopic images of the tested culture. Additionally, the study on lightly and darkly pigmented human melanocytes revealed that 100 µM of minocycline did not influence the viability of both cell lines after 48 h treatment. The cell viability was ca. 97% in control and treated cultures of melanocytes. Simultaneously, minocycline at the same concentration reduced the percentage of live melanoma cells to ca. 81%. The observations indicate a greater cytotoxic effect of minocycline on melanoma cells than on normal melanocytes.

Noticeable inhibition of melanoma cell proliferation was reflected in the disturbance of the cell cycle. The cell cycle profile of treated cultures was characterized in general by the rise of G_1_/S and G_2_-M/S ratio when compared to the control. However, the most significant arrest in the G_1_ phase was caused by minocycline in a concentration of 50 μM after 48 h. In turn, 100 μM of minocycline had the greatest influence on G_1_/S ratio after 24 h and on G_2_-M/S ratio for both tested treatment times. Interestingly, the changes in the level of G_1_/S ratio were the smallest for the highest concentration of the drug. On the other hand, 200 μM of minocycline most significantly increased the number of cells in the sub-G1 phase, which indicated a cytotoxic effect and DNA damage. The results indicate that minocycline, depending on concentration, shows a diverse effect on human melanoma cells. Lower concentrations of the tested drug are cytostatic, however 200 µM of minocycline reveals the cytotoxic effect. The observation was also confirmed by the analysis of DNA fragmentation as well as the estimation of the percentage of dead cells in the treated cultures. In all these cases the most significant lesions and defects were stated for melanoma cells exposed to the highest drug concentration. The results indicated that minocycline at a concentration of 200 µM induced the process of DNA fragmentation as well as causing damage to the cell membrane which was confirmed by the DAPI penetration in non-fixed cells.

The minocycline-induced increase in cell number in the G_1_ phase was also found for human ovarian carcinoma cells OVACAR-3 after 24 and 48 h treatment [47]. The study also showed that minocycline decreased the percentage of cells in S and G_2_-M phases, proportionally with concentration. In turn, the profile of changes differs from those observed in hepatocellular carcinoma cells (HCC) [39]. In this case, minocycline arrested cells in the S phase after 72 h and decreased the percentage of cells in the G_2_-M phase after 24 h and 48 h. At the same time, the number of cells in the G_1_ phase did not differ significantly. These findings suggest the influence of minocycline on the cell cycle could be different and the effect probably depends on the type of cancer cells. The observed inhibition of cancer cell proliferation by minocycline can be connected with downregulation of cell-cycle regulatory proteins, such as cyclin D, cyclin E and cyclin-dependent kinases 1, 2 and 4, as well as with upregulation of a cyclin-dependent kinase inhibitor—protein p27 [39,48].

Apoptosis is a mechanism of programmed and natural cell death that plays an important role in the development and homeostasis of living organisms. Moreover, the process is considered effective in terminating and controlling cancer cell growth. For this reason, it has become a target of cancer therapy [49].

The annexin V assay was the first test we conducted to confirm the minocycline-induced apoptosis process in human melanoma cells. The presented results showed that minocycline in the concentrations of 100 µM and 200 µM significantly increased annexin V positive cells. Apoptosis is characterized by several morphologic changes of cells, such as chromatin condensation, nuclear fragmentation, and reduction of cell volume, which are preceded and triggered by many complex and connected molecular, biochemical and metabolic processes [50,51]. The induction of apoptosis occurs as a result of the activation of the intrinsic or extrinsic pathway [52]. The intrinsic pathway is evoked with the participation of mitochondria and related proteins, especially belonging to the BCL-2 family. Moreover, numerous other factors, such as growth factor deficit, DNA-damage, oxidative stress, increased intracellular level of calcium ions, or microtubule disruption, can activate the pathway [49,53].

The presented results show that minocycline induces changes in human melanoma cells, characteristic of the intrinsic pathway of apoptosis. The drug decreased the intracellular level of reduced thiols—one of the most important non-enzymatic antioxidants. The effect was proportional to the concentration of minocycline and the time of incubation. The thiol molecules (e.g., glutathione) are a well-known marker of oxidative stress [54]. The reduced form of glutathione (GSH) reacts with harmful reactive oxygen species, reduces them, and at the same time dimerizes to the oxidized form (GS-SG). Thus, the observed increase in cells with a low level of reduced thiols indicates minocycline-induced oxidative stress.

The reduction of mitochondrial membrane potential in melanoma cells treated with minocycline is another hallmark indicating the intrinsic pathway of apoptosis. The drug in concentrations of 100 µM and 200 µM increased the number of cells with depolarized mitochondria up to ca. 85% after 48 h incubation. A study conducted by Suzuki-Karasaki et al. [55] showed a positive loop between the depolarization of the mitochondrial membrane and accumulation of mitochondrial reactive oxygen species. The authors proved that depolarization of the mitochondrial membrane could be potentiated by the accumulation of mitochondrial reactive oxygen species and, on the other hand, membrane-depolarizing agents caused the rise of mitochondrial ROS production. Thus, the observed activities of minocycline, involving stimulation of oxidative stress and mitochondrial membrane depolarization, could be interrelated.

The extrinsic pathway is stimulated by extracellular signals—deaths ligands, including Fas ligand (Fas-L), TNF-related apoptosis-inducing ligand (TRAIL) and tumor necrosis factor (TNF). The signals lead to the activation of cysteine proteases, i.e., caspases. The proteolytic activity of the enzymes is responsible e.g., for observed hallmarks of apoptosis, such as membrane blebbing or DNA fragmentation. The type of activated caspase is linked with the type of apoptotic pathway or with the stage of apoptosis. Extrinsic cell death is connected with caspase 8, whereas caspase 9 is characteristic of the intrinsic pathway [56]. Activation of initiator caspases (e.g., caspase 8 or 9) from inactive procaspase monomers, is a result of dimerization process. In turn, apoptotic effector caspases, including caspases 3 and 7, are produced as inactive procaspase dimers, which are cleaved and activated by initiator caspases [57].

The results suggesting the activation of apoptosis by minocycline prompted us to examine the contribution of the extrinsic and intrinsic pathways. For this purpose, the activity of caspases 8 and 9 was evaluated. The assay showed that minocycline can activate both of the proapoptotic pathways in human melanoma cells. The observed effect was proportional to the incubation time as well as to the drug concentration. Finally, the minocycline-induced apoptosis of melanoma cells was confirmed by the evaluation of effector caspases 3/7. The results of the test were similar to the other caspase tests, in general. The number of cells with active caspase 3/7 increased proportionally with the concentration of minocycline and the time of treatment. Stimulation of caspase activity by minocycline was also confirmed in other studies on cancer cells. Liu et al. [58] reported that minocycline induced autophagy of glioma cells whose mechanism included the activation of caspase 3. Moreover, the work of Song et al. [59] indicated that minocycline evoked the DNA damage, the release of cytochrome c from mitochondria which, in turn, triggered activation of effector caspase 7 in acute myeloid leukemia cells. Interestingly, the influence of the tested drug on caspase activity in non-cancer diseases can be different. The studies on cardiac ischemia/reperfusion (I/R) injury [60], retinal photic injury [61] and a cellular model of Huntington’s disease [62] showed the drug inhibited activation of caspases. These findings suggest that minocycline, probably in a drug concentration and disease-dependent manner, modulates variously caspase activity.

## 4. Conclusions

To summarize, the presented results indicate effective anti-melanoma activity of minocycline. The treatment of human melanoma cells inhibited their proliferation, disturbed cell cycle, and significantly changed cell morphology. The performed tests suggested that the lowest concentration of the drug showed a cytostatic activity, whereas 100 µM and 200 µM of minocycline appeared to be more cytotoxic. Moreover, it was found that the anti-melanoma effect of minocycline is connected with the induction of apoptosis. The drug caused a decrease in mitochondrial membrane potential as well as an increase in cells with a low level of reduced thiols. Finally, the apoptosis was confirmed by observed induction of caspases 8, 9, and 3/7 activity as well as by the rise of the number of annexin V-positive cells. Concerning many current problems with melanoma treatment, minocycline seems to be a drug with promising and potent anti-cancer properties that could be useful in therapy.

## 5. Materials and Methods

### 5.1. Chemicals and Reagents

Minocycline hydrochloride, C_23_H_27_N_3_O_7_ x HCl, amphotericin B, penicillin, Phalloidin-Atto 565, glycine, and Triton X-100 solution were obtained from Sigma-Aldrich Inc. (Taufkirchen, Germany). The growth medium M-254 as well as a human melanocyte growth supplement-2 (HMGS-2), and trypsin inhibitor were acquired from Cascade Biologics (Portland, OR, USA). Neomycin was purchased from Amara (Kraków, Poland). The growth medium RPMI 1640, fetal bovine serum, and trypsin/EDTA were obtained from Cytogen (Zgierz, Poland). Tyrosinase monoclonal antibody, secondary antibody—Alexa Fluor 488, SYTO Deep Red Nucleic Acid Stain, and Dulbecco’s phosphate-buffered saline (DPBS) were purchased from Thermo Fisher Scientific (Waltham, MA, USA). Cell Proliferation Reagent WST-1 was acquired from Roche GmbH (Mannheim, Germany). Solution 3 (1 μg/mL DAPI, 0.1% triton X-100 in PBS), Solution 5 (400 μg/mL VitaBright-48™, 500 μg/mL propidium iodide, 1.2 μg/mL acridine orange in DMSO), Solution 7 (200 μg/mL JC-1), Solution 8 (1 μg/mL DAPI in PBS), Solution 15 (500 μg/mL Hoechst 33342), Solution 16 (500 μg/mL propidium iodide), Via-1-Cassettes™ (acridine orange and DAPI fluorophores), NC-Slides™ A2, and A8 were obtained from ChemoMetec (Allerod, Denmark). Annexin V-CF488A conjugate as well as Annexin V binding buffer were purchased from Biotium (Fremont, CA, USA). Caspase 3/7 Assay Kit, Caspase 8 Assay Kit, and Caspase 9 Assay Kit were purchased from ImmunoChemistry Technologies (Bloomington, MN, USA). The remaining chemicals were acquired from POCH S.A. (Gliwice, Poland) or Sigma-Aldrich (Germany).

### 5.2. Cell Cultures

Human skin melanotic melanoma cell line, COLO 829, was acquired from ATCC (CRL-1974™, USA). The cells were cultured in RPMI 1640 medium with the addition of inactivated fetal bovine serum to a final concentration of 10%, penicillin (100 U/mL), neomycin (10 µg/mL) and amphotericin B (0.25 µg/mL). Human epidermal melanocytes, neonatal, lightly (HEMn-LP) and darkly pigmented (HEMn-DP) were purchased from Cascade Biologics, UK. The experiments were performed on melanocytes from passages 6 to 10. The growth medium was supplemented with a human melanocyte growth supplement-2 (HMGS-2) as well as antibiotics: penicillin (100 U/mL), neomycin (10 μg/mL) and amphotericin B (0.25 μg/mL). Cells were grown in a 5% CO_2_ incubator CB 160 (BINDER, Tuttlingen, Germany) at 37 °C with 5% relative humidity.

### 5.3. Screening Test of Cells Viability

The viability of human melanoma cells COLO 829 was evaluated by the WST-1 assay. Chemically, WST-1 is a slightly red tetrazolium salt, which is reduced to dark red formazan dye by cell mitochondrial dehydrogenases. The principle of the assessment is that the amount of produced formazan correlates with the number of viable cells. Melanoma cells in the amount of 2.5 × 10^3^ cells per well were seeded in a 96-well microplate in RPMI 1640 growth medium and placed in the incubator for 24 h. Next, the medium was replaced by minocycline solutions in concentrations ranging from 0.1 µM to 400 µM. After 21 h, 45 h, and 69 h of the treatment, 10 μL of WST-1 solution was added to each well, and the incubation was continued for 3 h. The absorbance measurement was made using microplate reader Infinite 200 PRO (TECAN, Männedorf, Switzerland) at 440 nm and 650 nm as a reference wavelength. The controls were also normalized to 100% for each assay and the treatments were expressed as the percentage of the controls.

### 5.4. Cell Treatment

Directly before the study, human melanoma cells as well as lightly and darkly pigmented melanocytes were seeded in T-75 flasks (2 × 10^6^ cells per flask). The treatment with minocycline was started 24 h and 48 h after seeding for melanoma cells and melanocytes, respectively. Afterwards, the cells were harvested by the use of trypsin, centrifuged and suspended in the growth medium.

### 5.5. The Evaluation of Cell Number and Cell Viability

Cell counting and the estimation of cell viability were made using NucleoCounter^®^ NC-3000™ (ChemoMetec)—a fluorescent imaging cytometer. The method is based on staining of non-fixed cells with acridine orange (detection of total cells population) and DAPI (detection of dead cells). During tests the cells, suspended in the growth medium, were loaded into Via1-Cassettes™ (ChemoMetec) containing the stains and then immediately analyzed using the Cell Viability and Cell Count Assays protocol by an NC-3000 image cytometer. The instant and rapid analysis means that DAPI penetrates a damaged and permeable cell membrane, hence it only stains non-viable cells in this condition.

### 5.6. Cell Morphology Assessment

The assessment of the influence of minocycline on human melanoma cell abundance and morphology was carried out after 24 h-long treatment. The cells were cultured in sterile cover slips placed in Petri dishes. After the exposure to minocycline in concentrations of 50 µM, 100 µM, and 200 µM, the cells were fixed with paraformaldehyde (4%), washed three times with PBS, and permeabilized with 0.1% Triton X-100 solution. In the next step, cells were treated with 0.25% glycine solution for 30 min and then incubated overnight with the primary tyrosinase monoclonal antibody. Afterward, the tested cells were washed three times with PBS and stained with 1 µM of SYTO (specific for DNA), 0.6 µM of Phalloidin–Atto 565 (specific for actin filaments), and 10 µg/mL of the secondary antibody conjugated with Alexa Fluor 488 (tyrosinase).

After the staining procedure, the cover slips were mounted onto microscopic glass slides. The samples were imaged by means of the laser confocal microscope Nikon Eclipse Ti-E A1R-Si and Nikon NIS Elements AR software.

### 5.7. Cell Cycle Assay

The cell cycle analysis of tested cells was performed using the NucleoCounter^®^ NC-3000™ fluorescent imaging cytometer (ChemoMetec). The method principle is based on measurements of the DNA content within cell population. The evaluation of the cell cycle was made after 24 h and 48 h-long treatments. The cells in an amount of 1 × 10^6^ were suspended in 0.5 mL PBS and fixed with 4.5 mL of 70% cold ethanol for at least 12 h at 0–4 °C. Before analysis, the cells were centrifuged, the ethanol was removed and the cell pellets were resuspended in PBS and centrifuged again for 5 min at 500× *g*. Next, the obtained cells pellets were stained by suspension in 0.5 mL of Solution 3 (1 μg/mL DAPI, 0.1% triton X-100 in PBS) and incubation for 5 min at 37 °C. Finally, the stained cells were loaded into 8-chamber NC-Slides A8™ and analyzed using the Fixed Cell Cycle-DAPI Assay protocol by the NC-3000 image cytometer. The obtained DNA content histograms were used to demarcate different phases of cell cycle in tested samples. The results were also presented as the relative quantity ratio: G_1_/S and G_2_-M/S.

### 5.8. Annexin V Assay

The annexin V assay was used to detect the process of cell apoptosis. The test principle is based on the high affinity of protein annexin V to phosphatidylserine whose translocation to the outer membrane layer occurs already in the early stage of apoptosis. The assay and its analysis were performed using the fluorescence image cytometer NucleoCounter^®^ NC-3000™ (ChemoMetec). In brief, 3.0 × 10^5^ melanoma cells were suspended in 100 μL of Annexin V binding buffer with 2 µL of Annexin V-CF488A conjugate (FITC-labeled annexin V) and 2 µL of Solution 15 (Hoechst33342 stains total population). In the next step, the cells were incubated for 15 min at 37 °C and subsequently centrifuged at 400× *g* for 5 min. Following the removal of the supernatant, the cell pellets were resuspended in 300 µL of Annexin V binding buffer and centrifuged twice under the conditions described above. Eventually, the cell pellets were resuspended in 100 µL of Annexin V binding buffer and 2 µL of Solution 16 (propidium iodide stains late apoptotic and necrotic cells) was added. The prepared samples were analyzed immediately using 8-chamber NC-Slides A8™ and the Annexin V Assay protocol. The obtained scatter-plots were used to demarcate the percentage of healthy cells and early and late apoptotic cells, respectively.

### 5.9. Analysis of Intracellular Thiol Level

The estimation of intracellular thiol level was performed using the fluorescence imaging cytometer NucleoCounter^®^ NC-3000™ (ChemoMetec). The analysis uses a specific dye, i.e., VitaBright-48™ (Solution 5) to stain cells with a high level of reduced thiols, such as GSH. The tested cells after the treatment with minocycline were harvested and counted using the NC-3000 image cytometer. Subsequently, 10 µL of Solution 5 was added into 190 µL of the cells suspension (2 × 10^6^ cells/mL). Next, the stained cells were loaded into 8-chamber NC-Slides A8™ and measured using the Vitality (VB-48) Assay protocol in the NC-3000 image cytometer. The obtained VB-48™ intensity histograms were used to demarcate the healthy and low vitality subpopulations of the tested samples.

### 5.10. DNA Fragmentation Assay

The estimation of DNA fragmentation in the human melanoma cells treated with minocycline was performed using the fluorescent imaging cytometer NucleoCounter^®^ NC-3000™ (ChemoMetec). The assay is based on the detection of cells with high molecular weight fragmented DNA, stained with DAPI. After the treatment with minocycline, the cells were harvested, counted, suspended in PBS (2 × 10^6^ cells/mL), fixed with 70% cold ethanol for at least 12 h at 0 °C–4 °C, and centrifuged, in sequence. The obtained cell pellets were resuspended in PBS, centrifuged again for 5 min at 500× *g* and next resuspended in 0.5 mL of Solution 3, containing 1 μg/mL DAPI and 0.1% triton X-100 in PBS. Afterward, stained cells were incubated for 5 min at 37 °C and loaded into 8-chamber NC-Slides A8™ to be analyzed. The obtained histograms were used to demarcate the subpopulation of the tested cell culture with fragmented DNA.

### 5.11. Mitochondrial Potential Assay

The assessment of mitochondrial transmembrane potential (ΔΨm) was made by the use of the fluorescence image cytometer NucleoCounter^®^ NC-3000™ (ChemoMetec). The principle of the method is related to the accumulation of fluorescent cationic dye JC-1 in the mitochondria in a potential-dependent manner. A high concentration of the dye inside the mitochondria, observed in healthy cells, favors its aggregation to the red fluorescent form. In turn, in apoptotic cells, JC-1 is localized in the cytoplasm and emits green fluorescence. After the treatment, 1.0 × 10^6^ cells were suspended in 12.5 μL of Solution 7 (JC-1) and next incubated for 10 min at 37 °C. The stained cells were then centrifuged at 400× *g* for 5 min and washed twice with PBS. The obtained cell pellets were resuspended in 0.25 mL of Solution 8 (DAPI) and analyzed immediately using 8-chamber NC-Slides A8™ and the Mitochondrial Potential Assay protocol. The results presented in the form of scatter-plots were used to demarcate the percentage of polarized/healthy cells and depolarized/apoptotic cells.

### 5.12. Caspase Activity Assay

The measurement of caspase activity is based on the use of fluorochrome-labelled inhibitor of caspases (FLICA reagent). The inhibitor passes through the cell membrane and binds to the cysteine residue of the active caspase heterodimer. The unbound inhibitor diffuses out of the cell and is washed away during preparation of the samples. In the first step, 5 µL of diluted FLICA reagent and 2 µL of Hoechst 33342 (stains total cell population) was added to 93 µL the cell suspension (4 × 10^6^ cells/mL). Then, the cells were incubated for 60 min at 37 °C. Afterwards, the samples were washed with 400 µL of apoptosis wash buffer and centrifuged, in the two consecutive cycles. Finally, the cells were resuspended in 100 μL apoptosis wash buffer supplemented with 10 μg/mL Solution 16 (propidium iodide—which stains necrotic cells) and analyzed immediately in the fluorescence image cytometer NucleoCounter^®^ NC-3000™ (ChemoMetec) using 2-chamber NC-Slides A2™ and the Caspase Assay protocol.

### 5.13. Statistical Analysis

Statistical analysis of the results was performed using GraphPad Prism 6.01 Software. In all experiments mean values of at least three separate experiments performed in triplicate (*n* = 9) ± standard deviation of the mean (SD) were calculated. The results were analyzed statistically by one-way ANOVA as well as Dunnett’s comparison test. The Kolmogorov–Smirnov test checked the compliance of the distribution results and the Brown–Forsythe test checked whether the variances of the compared groups met the homogeneity assumption. In all cases, the statistical significance was found for the *p*-value to be lower than 0.05.
